# The Influence of an Obesogenic Diet on Oxysterol Metabolism in C57BL/6J Mice

**DOI:** 10.1155/2014/843468

**Published:** 2014-02-05

**Authors:** Joshua S. Wooten, Huaizhu Wu, Joe Raya, Xiaoyuan Dai Perrard, John Gaubatz, Ron C. Hoogeveen

**Affiliations:** ^1^Division of Atherosclerosis and Vascular Medicine, Department of Medicine, Baylor College of Medicine, Houston, TX 77030, USA; ^2^Department of Kinesiology and Health Education, Southern Illinois University Edwardsville, Campus Box 1126, Edwardsville, IL 62026-1126, USA

## Abstract

Our current understanding of oxysterol metabolism during different disease states such as obesity and dyslipidemia is limited. Therefore, the aim of this study was to determine the effect of diet-induced obesity on the tissue distribution of various oxysterols and the mRNA expression of key enzymes involved in oxysterol metabolism. To induce obesity, male C57BL/6J mice were fed a high fat-cholesterol diet for 24 weeks. Following diet-induced obesity, plasma levels of 4**β**-hydroxycholesterol, 5,6**α**-epoxycholesterol, 5,6**β**-epoxycholesterol, 7**α**-hydroxycholesterol, 7**β**-hydroxycholesterol, and 27-hydroxycholesterol were significantly (*P* < 0.05) increased. In the liver and adipose tissue of the obese mice, 4**β**-hydroxycholesterol was significantly (*P* < 0.05) increased, whereas 27-hydroxycholesterol was increased only in the adipose tissue. No significant changes in either hepatic or adipose tissue mRNA expression were observed for oxysterol synthesizing enzymes 4**β**-hydroxylase, 27-hydroxylase, or 7**α**-hydroxylase. Hepatic mRNA expression of SULT2B1b, a key enzyme involved in oxysterol detoxification, was significantly (*P* < 0.05) elevated in the obese mice. Interestingly, the appearance of the large HDL_1_ lipoprotein was observed with increased oxysterol synthesis during obesity. In diet-induced obese mice, dietary intake and endogenous enzymatic synthesis of oxysterols could not account for the increased oxysterol levels, suggesting that nonenzymatic cholesterol oxidation pathways may be responsible for the changes in oxysterol metabolism.

## 1. Introduction

During obesity and insulin resistance, increased adipocyte lipolysis paired with an inability of the fat cell to expand further to store lipid promotes excess ectopic lipid deposition (i.e., lipotoxicity), a major contributor to the development of cardiometabolic diseases [1]. Lipotoxicity generally refers to cellular injury and death caused by free fatty acids (FFA) and related-lipid metabolites [1]. In addition, cholesterol and its oxygenated species (oxysterols) add further to the complexity of the identification of lipotoxic species that affect the regulation of lipid and glucose metabolism, inflammation, endoplasmic reticulum (ER) stress, apoptosis, and necrosis [2]. The role of FFA in lipotoxicity has been extensively studied; however, it remains to be elucidated what role oxysterols may play in obesity and subsequent tissue lipotoxicity.

During hepatic lipotoxicity, the hepatic pools of FFA and cholesterol increase significantly, thereby promoting a cytotoxic environment [1]. The bulk of hepatic FFA are either esterified to glycerol to form triglycerides or oxidized in peroxisomes and mitochondria. However, increased FFA oxidation can lead to the accumulation of reactive oxygen species (ROS) due to a reduced endogenous antioxidant capacity observed with nonalcoholic fatty liver disease [1].

Cholesterol is essential for proper cell function as a key component of cellular membranes and precursor of steroid hormones [3]; however, excess cholesterol can lead to deregulation of cholesterol metabolism, which is believed to be an underlying pathology in the development of atherosclerosis, diabetes, stroke, and cancer [3]. In mammals, the liver is a key organ involved in the removal of excess cholesterol through its conversion into bile acids. Cholesterol degradation involves a number of monooxygenation reactions, which are catalyzed by specific enzymes called cytochromes P450 (CYPs). During normal hepatic cholesterol degradation, cholesterol can be enzymatically converted to 7*α*-hydroxycholesterol by 7*α*-hydroxylase (CYP7A1), which is the rate-limiting step in bile acid formation. Other CYP enzymes such as 27-hydroxylase (CYP27A1) and 4*β*-hydroxylase (CYP3A4 in human and CYP3A11 in mouse) have been shown to catalyze the formation of the major oxysterol species 27-hydroxycholesterol and 4*β*-hydroxycholesterol, respectively. Alternatively, cholesterol can be subject to nonenzymatic autooxidation, which leads to the formation of a number of cholesterol oxidation products, including 7-ketocholesterol, 7*α*-hydroxycholesterol, 7*β*-hydroxycholesterol, 5,6*α*-epoxycholesterol, and 5,6*β*-epoxycholesterol. Although it is generally believed that under normal physiological conditions tissue levels of oxysterols are largely a reflection of endogenous enzymatic synthesis, it is currently not clear what role oxysterols derived from the action of ROS or dietary intake play in oxysterol metabolism in the obese state.

Data from a growing number of research studies suggest that oxysterols play an important role in the regulation of lipid metabolism [4]. However, oxysterols have been shown to be involved in the development of atherosclerosis, apoptosis, and inflammation [5]. There is a paucity in data on tissue distributions of oxysterols, particularly during different pathological states in both human and animal models. Matsui et al. [6] were one of the first to characterize that 7*β*-hydroxycholesterol and 7-ketocholesterol were significantly elevated in lean, diabetic rat hearts in comparison to controls. Yoshioka et al. [7] observed similar increases in 7*β*-hydroxycholesterol and 7-ketocholesterol, as well as 7*α*-hydroxycholesterol in lean, diabetic rat heart, kidney, and liver tissues. Diabetes has been associated with a state of increased oxidative stress [8]. Since 7*β*-hydroxycholesterol and 7-ketocholesterol are largely generated via nonenzymatic pathways involving ROS, it is plausible that the increased oxidative stress can partly explain their findings. It is important to note that diabetes in both of these studies was induced by streptozotocin, which may yield different changes in tissue oxysterols than what may be observed naturally during obesity-induced type 2 diabetes.

It remains to be elucidated to what extent oxysterol metabolism is altered during diet-induced obesity. Therefore, the purpose of this study was to determine the effects of obesity induced by a high fat-cholesterol diet on oxysterol levels in plasma, liver, and adipose tissue. In addition, we measured the expression of oxysterol synthesizing enzymes CYP7A1, CYP3A11, and CYP27A1 mRNA in liver and adipose tissue. Furthermore, we examined the expression of SULT2B1b mRNA, a cytosolic sulfotransferase that is believed to sulfonate oxysterols to prevent their cytotoxic effects. Tissue lipid composition and circulating lipoprotein composition were quantified to describe the severity of the dyslipidemia in these mice after 24 weeks of a high fat-cholesterol (HFC) diet.

## 2. Methods and Materials

### 2.1. Animals and Diet

Male C57BL/6J mice (Jackson Laboratory, Bar Harbor, ME) were used and divided into two dietary groups (normal control diet or HFC diet) at 8 weeks of age. The normal control diet (ND) consisted of 5.0% w/w fat (12.0% of kcal from fat) and 0.03% w/w cholesterol (LabDiet #5053, Brentwood, MO) and the HFC contained 21.0% w/w fat (41% of kcal from fat) and 0.15% w/w cholesterol (Dyets Inc., Bethlehem, PA). Mice were fed *ad libitum* and were kept on a 12 hr light/dark cycle. Following 24 weeks of feeding, mice were sacrificed, body weight was measured, and fasting blood (12 hr) was collected by heart puncture and treated with EDTA prior to separation by low speed centrifugation (1500 g, 15 min, 4°C). Liver and perigonadal fat pads were harvested, weighted, and immediately frozen in liquid N_2_. Plasma and tissues were stored at −80°C for further analysis. This study was approved by the Institutional Animal Care and Use Committee of Baylor College of Medicine and all experimental procedures were in accordance with institutional guidelines.

### 2.2. Measurement of Plasma Lipoprotein Lipid Composition

Lipoproteins were isolated from 12 hr fasted plasma (500 *μ*L) by density gradient ultracentrifugation according to previously published method [9]. Isolated lipoprotein samples (500 *μ*L) were subjected to fast performance liquid chromatography (FPLC) gel filtration using two Superose 6 10/300 GL columns. Individual 500 *μ*L fractions were pooled into VLDL (fractions 15–19), LDL (fractions 21–24), LDL/HDL_1_ (fractions 25–27), and HDL (fractions 29–34) fractions and then were assayed for concentrations of total cholesterol, triglyceride, phospholipid (Wako Chemicals, Richmond, VA), and protein (Bio-Rad, Hercules, CA). HDL_1_ was verified using Western blot of apo AI and apo E proteins. Prior to immunoblot analysis, pooled FPLC fractions for HDL_1_ were concentrated using centrifugal filters (UFC501096, Millipore, Billerica, MA). Following the determination of total protein concentration (Q33210, Invitrogen, Carlsbad, CA), 10 *μ*g of total protein was electrophoresed on 4–20% tris-glycine gels (#EC6028, Invitrogen, Carslbad, CA), transferred to nitrocellulose membranes, and probed for apo AI (1 : 2000; ab20355) and apo E (1 : 4000; ab20874). To identify the possible presence of small, dense LDL particles 10 *μ*g of total protein was blotted for apo B (1 : 4000; ab20355). All primary antibodies and HRP-conjugated secondary antibodies were purchased from Abcam, Inc. (Cambridge, MA). Equal amounts of total protein were loaded for both ND and HFC groups in each lane. Bands were revealed using SuperSignal West Femto Chemiluminescence Substrate (Thermo-Fisher Scientific, Rockford, IL).

### 2.3. Lipid and Oxysterol Extraction and Quantitation

Tissues were weighed (30–50 mg), finely ground to a powder under liquid N_2_, and incubated overnight at room temperature in 1 mL of hexane: isopropanol (3 : 2, v/v) with 0.1% (w/v) butylated hydroxytoluene (BHT). Samples are centrifuged (15 min at 4000 g) and the supernatant was transferred to a clean tube and evaporated under N_2_. The dried lipid extract was weighed to determine the total lipid content of the tissues [10]. The lipid extract was resuspended in 1 mL of isopropanol. The protein pellet was resuspended in 0.2 N NaOH and incubated at room temperature overnight prior to performing any analytical measurements as described [11].

Total cholesterol and triglyceride assays were performed on the liver and adipose tissue lipid extracts using standard colorimetric assay kits (Wako Chemicals, Richmond, VA). From the protein extract, protein concentrations were measured using a standard Lowry assay (Bio-Rad, Hercules, CA).

Oxysterol measurements were performed on diet, plasma, liver, and adipose tissue by gas chromatography-mass spectrometry (GC-MS) using a modified isotope dilution method described by Dzeletovic et al. [12]. Lipids were extracted from the chow and plasma using a modified Folch technique [13] with the addition of 0.1% (w/v) BHT and 10 *μ*L of internal standard to each sample. The internal standard contained seven deuterium-labeled oxysterols (Avanti Polar Lipids, Alabaster, AL), 7*α*-hydroxycholesterol, 7*β*-hydroxycholesterol, 7-ketocholesterol, 4*β*-hydroxycholesterol, 25-hydroxycholesterol, 27-hydroxycholesterol, 5*α*,6*α*-epoxycholesterol, and 5*β*,6*β*-epoxycholesterol at a concentration of 20.0 ng/mL for each standard. Diet samples (~200 mg from five individual pellets) were incubated at room temperature for 1 hr to ensure complete lipid separation followed by addition of 2 mL 0.9% (w/v) NaCl. Due to small plasma volumes collected from the mice, mouse plasma samples were pooled (2-3 mice per 500 *μ*L volume per sample) prior to Folch extractions. Both diet and plasma samples were centrifuged (10 min at 1500 g), followed by extraction of the lower organic lipid phase and dried under N_2_. To the tissue lipid extract (250 *μ*L), 10 *μ*L of internal standard was added and then evaporated under N_2_.

Dried plasma, tissue, and diet lipid extracts were subjected to hydrolysis for 2 hr following addition of 1 mL PBS and 2 mL 0.5 M KOH. To terminate hydrolysis, 1 mL of 1 M HCl was added followed by 2 mL of hexane with 0.1% BHT. Following centrifugation, the upper organic phase was extracted and evaporated under N_2_. Prior to solid-phase separation of cholesterol and oxysterols, 1 mL of toluene was added to each sample. Solid-phase extraction was performed using 200 mg solute silica cartridges (Agilent Technologies, Inc., Santa Clara, CA). Cholesterol was eluted from the cartridges using 12–18 mL of 0.5% (v/v) isopropanol in hexane followed by elution of oxysterols with 8 mL 60% (v/v) isopropanol in hexane. The oxysterol fraction was evaporated under N_2_ and converted to trimethysiyl ether by adding 1 : 1 (v/v) pyridine : bis(trimethylsilyl)trifluoroacetamide and heated for 30 min at 60°C. Following evaporation under N_2_, the residue was dissolved in 100 *μ*L decane and transferred to autosampler vials.

Oxysterol concentration was measured by GC-MS on an Agilent GC6890N equipped with a DB-5MS capillary column, connected to an Agilent 5973 inert mass selective detector (Agilent Technologies, Inc., Santa Clara, CA). The mass spectrometer was operated in the selected ion monitoring mode, and the ions used for analysis (*m/z*) were as follows: 7*α*-hydroxycholesterol, 456.5 *m/z*; [^2^H_7_] 7*α*-hydroxycholesterol, 463.6 *m/z*; [^2^H_7_] 7*β*-hydroxycholesterol, 463.6 *m/z*; 7*β*-hydroxycholesterol, 456.5 *m/z*; [^2^H_7_] 7-ketocholesterol, 479.5 *m/z*; 7-ketocholesterol, 472.0 *m/z*; 25-hydroxycholesterol, 131.0 *m/z*; [^2^H_3_] 25-hydroxycholesterol, 134.0 *m/z*; 27-hydroxycholesterol, 456.4 *m/z*; [^2^H_5_] 27-hydroxycholesterol, 461.5 *m/z*; 5,6*α*-epoxycholesterol, 474.5 *m/z*; [^2^H_7_] 5,6*α*-epoxycholesterol, 481.6 *m/z*; 5,6*β*-epoxycholesterol, 474 *m/z*; [^2^H_7_] 5,6*β*-epoxycholesterol, 481.6 *m/z*; 4*β*-hydroxycholesterol, 456.5 *m/z*; and [^2^H_7_] 4*β*-hydroxycholesterol, 463.6 *m/z*.

### 2.4. Quantitation of mRNA Expression

mRNA of CYP7A1, CYP27A1, CYP3A11, and SULT2B1b in mouse liver and adipose tissue was examined by quantitative reverse transcriptase polymerase chain reaction (qRT-PCR) using predesigned primers and probes (Applied Biosystems, Carlsbad, CA) as previously described [14].

### 2.5. Statistical Analysis

Data are presented as mean ± SD. Independent sample *t*-tests were used to identify significant differences between dietary groups for all measured variables. Simple correlations between diet, plasma, and liver and adipose tissues were performed using Pearson product correlations. All calculations were performed with SPSS v19.0 statistical software package (IBM, Armonk, NY). Statistical significance was set at *P* < 0.05.

## 3. Results

### 3.1. Body and Tissue Mass

The total body weight of the HFC group was significantly (*P* < 0.05) greater than the ND group (33.2 ± 5.2 g in ND group versus 49.0 ± 3.6 g in the HFC group; *n* = 10/group) following the 24-week diet intervention. Perigonadal fat pads (1.3 ± 0.5 g in ND group versus 3.2 ± 0.4 g in HFC group; *n* = 10/group) and liver (1.4 ± 0.3 g in ND group versus 3.9 ± 0.8 g in HFC group; *n* = 10/group) were significantly (*P* < 0.05) greater in the HFC group than the ND group.

### 3.2. Tissue Lipid Composition

**Figure 1:**
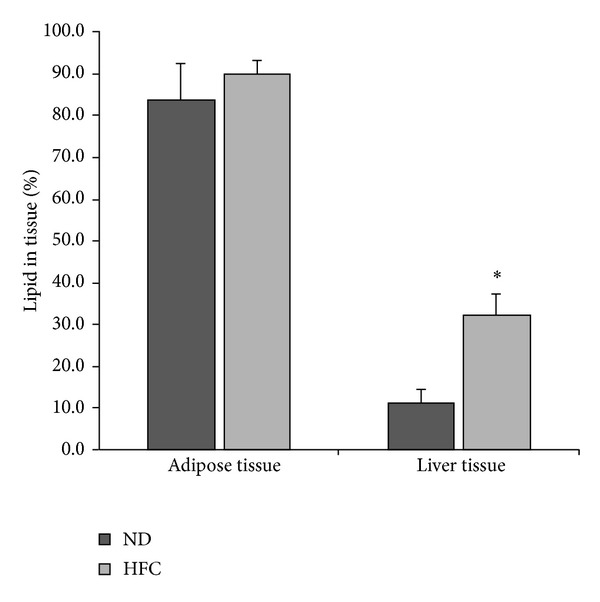
Percentage of lipid recovered from the adipose and liver tissue. No significant (*P* = 0.160) difference was observed between diet groups in the percentage of lipid present in the adipose tissue. Conversely, the HFC diet resulted in a significant (*P < 0.05) 3.3-fold greater percentage of lipid in the liver tissue when compared to the control group. ND: normal chow diet; HFC: high fat/high cholesterol diet.

The percentage of lipid by weight recovered from the adipose tissue and liver is shown in Figure 1. Despite a 6.8% greater percent lipid mass in the adipose tissue in the HFC group, no significant difference (*P* = 0.160) between the percentage of lipid mass was observed; however, after adjusting for cellular protein levels the HFC diet resulted in significantly greater cholesterol (*P* = 0.005) and triglyceride (*P* = 0.011) concentrations compared to ND (Table 1). The larger variability in the percent lipid mass of the control mice may partly explain the disassociation between the measured percent lipid mass and lipid concentrations in the adipose tissue. In contrast, the liver from the obese mice had a 3.3-fold greater (*P* < 0.001) percentage of lipid mass when compared to the ND group. In addition, the liver from the obese mice had significantly (*P* < 0.001) greater cholesterol and triglyceride concentrations than the ND.

### 3.3. Lipoprotein Lipid Composition

**Figure 2:**
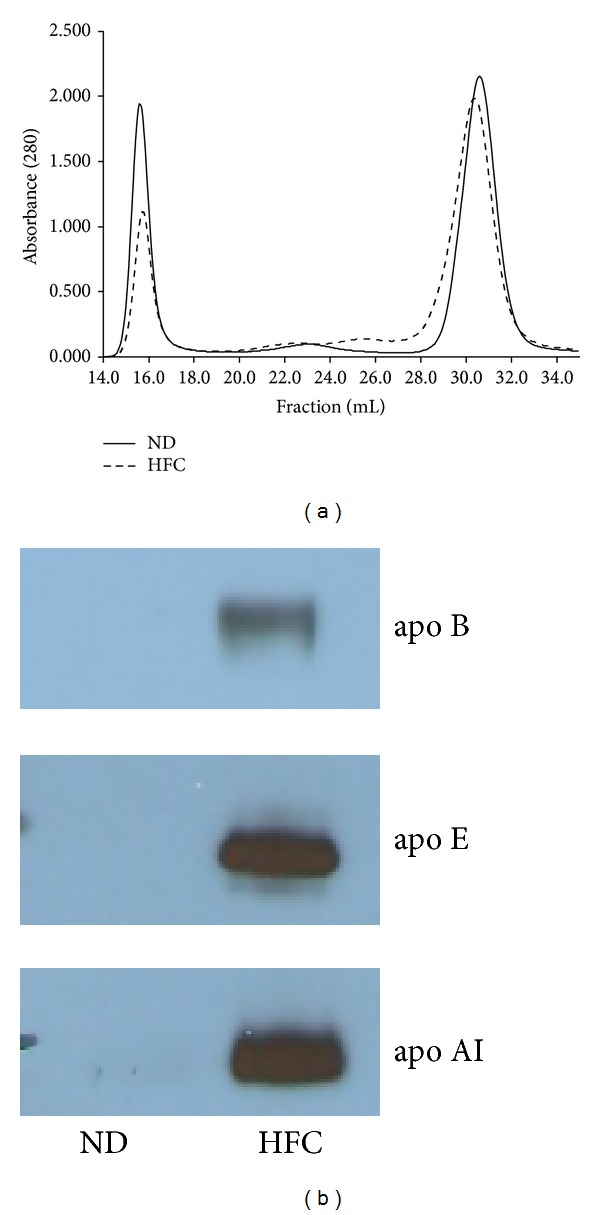
(a) FPLC lipoprotein profiles from mice after normal chow diet (solid line) and HFC diet (dashed line). Total lipoproteins were isolated by ultracentrifugation and lipoproteins were separated over two Superose 6 columns. Fractions 15–19 contain VLDL, 21–24 contain LDL, 25–27 contain LDL/HDL_1_, and HDL is found in fractions 29–34. (b) Western blot analysis of apo B, apo E, and apo AI in the pooled (six animals per group) FPLC fractions 25–27 (LDL/HDL_1_).

The mean lipoprotein-FPLC profile for each diet group is presented in Figure 2(a). The main differences in the lipoprotein profiles between the dietary groups were the greater ratio of HDL to VLDL particles and the appearance of a large HDL-like fraction in the mice on the HFC diet. The appearance of a large HDL-like lipoprotein fraction, also known as HDL_1_, has been previously reported in other mouse models of obesity. In leptin-deficeint (Lep^ob/ob^) and leptin-receptor-deficient (LepR^db/db^) mice, the development of obesity increases HDL without an increase in VLDL [15, 16], which has been shown to make these animals resistant to the development of atherosclerotic lesions [17]. Silver et al. [16, 18] showed that the increase in HDL and the formation of the larger HDL_1_ particles were caused by decreased hepatic HDL uptake. In the ob/ob and db/db mouse models, as well as SR-BI^−/−^, the HDL_1_ particles contain increased levels of apo E and apo A-I [19, 20]. The presence of HDL_1_ in HFC group was verified by elevated protein levels of apo AI and apo E in the HDL_1_ FPLC fraction (Figure 2(b)). Interestingly, there were elevated levels of apo B protein in the HFC group HDL_1_ FPLC fraction suggesting that this fraction was a mix of small dense LDL and large HDL_1_ particles. Therefore, we termed this lipoprotein fraction LDL/HDL_1_ consistent with previous reports. Even after concentrating the HDL_1_ FPLC fractions we did not find significant amounts of protein in the ND group and loaded the concentrated fraction just for comparison.

Lipid and protein composition of the pooled lipoprotein-FPLC fractions are presented in Table 2. When compared to the ND mice, the HFC group displayed significantly elevated cholesterol concentrations (uncorrected for protein concentration) in the VLDL, LDL, and HDL fractions. The triglyceride concentration was significantly elevated in the LDL, LDL/HDL_1_, and HDL fractions in the HFC group when compared to the ND group. Phospholipid concentration was significantly increased in the LDL, LDL/HDL_1_, and HDL fractions in the HFC group. Protein concentrations (Table 2) in the LDL and HDL fractions remained relatively unchanged following the 24-week HFC diet. In contrast, the protein concentration in the LDL/HDL_1_ fraction was significantly higher in the HFC group than the ND group, which was apparent by the LDL/HDL_1_ peak in the FPLC profile (Figure 2(a)); however, the VLDL protein concentration in the HFC group was significantly lower than the ND group. After correcting for protein concentration, cholesterol concentration was significantly elevated in the VLDL, LDL/HDL_1_, and HDL fractions of the HFC group. Triglyceride concentration was significantly increased in the LDL and HDL fractions of the HFC group, whereas phospholipid concentrations were significantly elevated in all the lipoprotein fractions of the HFC group.

### 3.4. Oxysterols in Diet, Plasma, and Tissues

No significant difference was observed for the majority of the measured dietary oxysterols between the ND and HFC chow (Table 3). Although the oxysterol content of both diets was extremely low, the 27-hydroxycholesterol concentration was significantly greater in the HFC diet than the control diet (control: 0.02 ± 0.01 ng/mg chow versus HFC: 1.95 ± 0.20 ng/mg chow; *n* = 5; *P* < 0.0001). Surprisingly, 7-ketocholesterol concentration was lower in the HFC diet compared to the control diet (ND: 0.84 ± 0.04 ng/mg chow versus HFC: 0.72 ± 0.08 ng/mg chow; *n* = 5; *P* < 0.05). No significant correlations were observed between the concentration of oxysterols in the normal and HFC chow and the plasma (*P* = 0.345), adipose tissue (*P* = 0.482), or liver tissue (*P* = 0.097) oxysterol levels.

Plasma, liver, and adipose tissue oxysterol concentrations are presented in Table 4. Except for 7-ketocholesterol and 25-hydroxycholesterol, plasma concentrations of all other oxysterols were significantly elevated in the HFC group by 2- to 10-fold. Plasma oxysterol levels were significantly correlated with adipose (*r* = 0.527, *P* = 0.036) and hepatic (*r* = 0.578, *P* = 0.019) oxysterol levels. In the adipose tissue, the HFC diet caused a significant 2.5- and 7.5-fold increase in 27-hydroxycholesterol and 4*β*-hydroxycholesterol, respectively. Furthermore, mean 5,6*α*-epoxycholesterol and 5,6*β*-epoxycholesterol levels were 2-fold higher in adipose tissue of mice on the HFC diet, although these increases did not reach significance due to substantial variance in these measurements. Interestingly, 25-hydroxycholesterol was 2.8-fold lower in the adipose tissue of the mice on the HFC diet; however, due to the large variance in both groups of mice the difference between diets was not significant. In contrast, only 4*β*-hydroxycholesterol was significantly elevated in the liver following the HFC diet. Hepatic 25-hydroxycholesterol was 3-fold higher in the HFC mice, but this difference was not significant due to the large variance in the HFC mice. The concentrations of all measured oxysterols were 2- to 24-fold higher in the adipose tissue compared to the liver. The combination of elevated adipose oxysterol concentrations and the large expansion of adipose tissue in the mice on the HFC diet suggests that adipose tissue is a key storage compartment for oxysterols in these obese mice.

### 3.5. Cytochrome P450 (CYP) Expression

**Figure 3:**
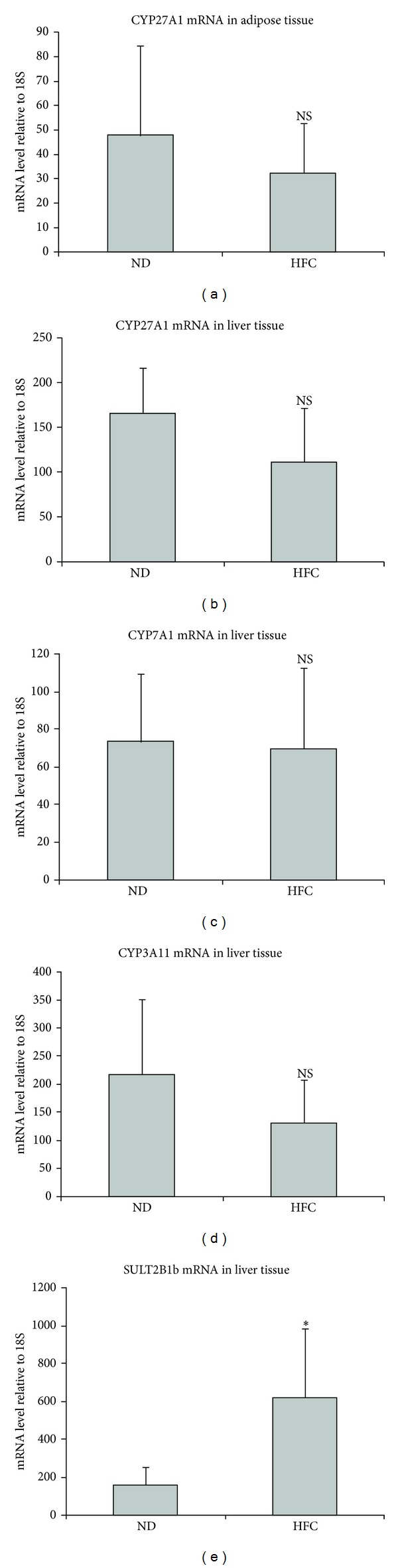
Cytochrome P450 27A1, 7A1, and 3A11 and SULT2B1b mRNA levels quantified by qPCR in adipose tissue and liver tissue of C57BL/6J mice (*n* = 9–13 mice per group). *Significantly (*P* < 0.05) different than control group. NS indicates not significantly different between groups.

Tissue specific CYP mRNA expression is illustrated in Figure 3. As expected, robust mRNA expression levels were detected for CYP3A11, CYP27A1, and CYP7A1 in the liver. Although hepatic mRNA expression levels of CYP27A1 and CYP3A11 tended to be lower in the mice on the HFC diet, no significant changes in mRNA levels were observed for CYP3A11, CYP27A1, or CYP7A1. In the adipose tissue, CYP7A1 and CYP3A11 mRNA expression were not detected. CYP27A1 mRNA expression was observed in adipose tissue but was not significantly different between dietary groups. In contrast, SULT2B1b mRNA expression was significantly elevated (>3-fold, *P* = 0.002) in the mouse liver following the HFC feeding. Interestingly, low levels of SULT2B1b mRNA expression were also detected in adipose tissue, but due to the large biological variance no significant differences were observed between groups (data not shown).

## 4. Discussion

### 4.1. Tissue and Lipoprotein Lipid Composition

In the present study, the HFC diet induced obesity in the mice as witnessed by the presence of increased total body mass, perigonadal fat pad mass, and liver mass. The adipose tissue was characterized by higher intracellular concentrations of cholesterol, as well as triglycerides. In the liver, the 3.3-fold increase in lipid mass was more pronounced and was matched by a 2.3-fold increase in cholesterol and 3.0-fold increase in triglyceride concentrations, characteristic of nonalcoholic fatty liver disease (NAFLD) [21]. These findings are in agreement with a previous report by Plummer and Hasty [21] who showed that C57BL/6 mice fed a custom butterfat diet (1.25% cholesterol, 19.5% fat, and 0.5% cholic acid) for 20 weeks had an approximate 2.1-fold increase in hepatic triglyceride in the obese mice compared to lean mice.

The lipoprotein profile and lipid composition of circulating lipoproteins were significantly modified by the 24-week HFC diet. We observed a significant increase in total cholesterol concentration of all lipoprotein fractions in the obese mice. The most substantial change was in the VLDL fraction where we observed an 11.5-fold increase in cholesterol. This change in VLDL lipid composition in C57BL/6 mice appears to be typical following a HFC feeding [21, 22]. LeBoeuf et al. [22] established that the increase in VLDL cholesterol following HFC diets was due to excess dietary cholesterol and not the dietary fat. Interestingly, increased triglyceride concentration was not observed in the VLDL fraction but was limited to the LDL and HDL fractions. The VLDL lipid composition in mice is distinctly different from human VLDL, which is triglyceride rich and cholesterol poor [23]. Moreover, unlike humans who transport cholesterol in LDL (65–85%), the mouse carries more than 85% of its plasma cholesterol in HDL [24, 25]. This phenomenon is not well understood; however, the difference between species is partly attributed to several factors such as the absence of cholesteryl ester transfer protein (CETP) in mouse plasma, hepatic lipase which is membrane-bound in humans and soluble in mice, reduced synthesis of apoB-100 in mice, and higher efficiency of apo E clearing remnant lipoproteins in mice [24]. These metabolic differences between species may also explain the presence of LDL/HDL_1_ particles that appears to be exclusive to obese mice [26]. We also detected the appearance of circulating LDL/HDL_1_ particles in our mouse model of diet-induced obesity. In the most commonly recognized mouse models of obesity, Lep^ob/ob^and LepR^db/db^, mice display elevated plasma cholesterol levels with a predominance of LDL/HDL_1_ particles [26]. Interestingly, Plummer and Hasty [21] showed that the LDL/HDL_1_ particle does not promote atherosclerosis and Lep^ob/ob^ mice are actually protected from diet-induced atherosclerosis when compared to C57BL/6J controls [15, 21], despite the development of obesity and hyperinsulinemia.

### 4.2. Oxysterol Metabolism

Mice on the HFC diet had markedly increased (2- to 10-fold) plasma oxysterol levels, with the exception of plasma 7-ketocholesterol and 25-hydroxycholesterol. The elevated circulating oxysterol levels are most likely a result of the diet-induced hypercholesterolemia rather than increased dietary oxysterol intake, since the oxysterol content of the HFC diet was extremely low and did not differ substantially from the control diet with exception of 27-hydroxycholesterol. Previous studies in humans have reported that the major oxysterols present in the circulation are from high-to-low concentration, 27-hydroxycholesterol, 24-hydroxycholesterol, 7*α*-hydroxycholesterol, and 4*β*-hydroxycholesterol [12, 27]. Interestingly, we found that from high-to-low concentration, 4*β*-hydroxycholesterol, 5,6*α*-epoxycholesterol, 5,6*β*-epoxycholesterol, and 7*α*-hydroxycholesterol were the major oxysterols present in the mouse plasma in both dietary groups. The apparent discrepancy between our data and those published previously may be due to species differences in lipoprotein and oxysterol metabolism, methodological differences in oxysterol measurements, or the fact that epoxycholesterols are not typically measured in most studies.

In the obese mice, 4*β*-hydroxycholesterol was elevated in the circulation as well as in the liver and adipose tissue. Bodin et al. [28] demonstrated that 4*β*-hydroxycholesterol is metabolized by the cytochrome P450 enzyme CYP3A4 in humans (CYP3A11 in mouse). In contrast to liver-X-receptor (LXR) regulating the activities of CYP7A1 and CYP27A1, CYP3A11 is regulated by pregnane-X-receptor via bile acid precursors [29], bile acids [30], and endogenous cholesterol derivatives such as 24(S),25-epoxycholesterol, 25-hydroxycholesterol, and 27-hydroxycholesterol [31]. In human plasma, 4*β*-hydroxycholesterol is present at relatively high concentrations, which has been primarily attributed to the slow elimination of this oxysterol from the circulation [27]. Compared to oxysterols 7*α*-hydroxycholesterol, 27-hydroxycholesterol, and 24-hydroxycholesterol for which half-lives have been determined to be 0.5 to 12 hr [32, 33], 4*β*-hydroxycholesterol has a half-life of approximately 2-3 days [34]. In the present study, 4*β*-hydroxycholesterol was the most prominent oxysterol in plasma, liver, and adipose tissue. We did not detect CYP3A11 mRNA expression in the adipose of C57BL/6 mice in either dietary treatment group. Interestingly, hepatic CYP3A11 mRNA expression was 40% lower in the obese mice, although this decrease was not statistically significant (*P* = 0.11). The lower mRNA expression of CYP3A11 may be due to negative feedback in response to elevated hepatic concentrations of 4*β*-hydroxycholesterol or certain bile acid precursors, including 27-hydroxycholesterol. These data suggest that the elevated 4*β*-hydroxycholesterol plasma and tissue concentrations in the obese mice are likely a result from decreased catabolic rate rather than an increased synthesis of 4*β*-hydroxycholesterol involving CYP3A11.

We did not find any evidence of altered hepatic CYP7A1 mRNA expression in the mice on the HFC diet. This seems in contradiction with a previous report by Gnerre and coworkers who showed that hepatic CYP7A1 mRNA expression was significantly elevated in C57BL/6 mice fed a low-fat standard chow diet supplemented with 2% cholesterol for 1 week [31]. However, it is important to point out that our HFC diet was a high-fat diet and contained only 0.15% (w/w) cholesterol, which was significantly lower than the diet used by Gnerre et al. Furthermore, in our study we did not find any significant effects of the HFC diet on 7*α*-hydroxycholesterol tissue levels. Since 7*α*-hydroxycholesterol can be formed via autooxidation of cholesterol, it is plausible that the elevated plasma levels of 7*α*-hydroxycholesterol in the HFC dietary treatment group are a result of increased cholesterol autooxidation due to the observed hypercholesterolemia in these mice.

Diet-induced obesity in C57BL/6 mice has been shown to cause an altered immune response observed by increased proliferation of splenic lymphocytes and the release of cytokines interferon- (IFN-)*γ* and interleukin- (IL-)4 [35]. The release of IFN-*γ*, a T helper- (Th-)1 cytokine that is central to regulating the obesity-induced adipose tissue inflammatory response [36, 37], upregulates the production of 25-hydroxycholesterol by increased 25-hydroxylase activity [38]. 25-Hydroxycholesterol is an important component of the innate immune response, as well as lipid metabolism by directly activating LXR [30]. Despite the enhanced inflammatory response previously shown in our diet-induced obese mouse model [14, 39], no significant changes in 25-hydroxycholesterol concentrations were observed in plasma or adipose and hepatic tissues following the 24-week dietary intervention.

27-Hydroxycholesterol has been demonstrated to be an alternative pathway for bile acid synthesis and efflux of cholesterol from macrophages [40]. Hirayama et al. [41] suggested that elevated 27-hydroxycholesterol concentrations may predict a reduced ability to maintain cholesterol homeostasis following high dietary cholesterol feedings and results in elevated circulating LDL-C. In the present study, we observed an increase in plasma 27-hydroxycholesterol, as well as an increase in the cholesterol content of LDL and HDL particles. In addition, unlike the control mice, the obese mice developed cholesterol rich LDL/HDL_1_ particles. This may be due in part to the fact that mice do not have CETP present to remove cholesteryl esters from HDL in exchange for triglyceride that can be later acted upon by hepatic lipase (HL). Therefore, elevated 27-hydroxycholesterol in mice may predict the presence of LDL/HDL_1_ particles, which is indicative of reduced HDL-mediated clearance of cholesterol.

In the mice on the HFC diet elevated plasma 27-hydroxycholesterol levels coincided with increased hepatic and adipose levels. The elevated 27-hydroxycholesterol tissue levels were not due to increased CYP27A1 mRNA expression, since both liver and adipose tissue CYP27A1 mRNA appeared to be slightly downregulated in the HFC group.

7*α*-Hydroxycholesterol, 7*β*-hydroxycholesterol, and 7-ketocholesterol are the product of decomposition of 7-hydroperoxycholesterol, which is formed by the autooxidation of cholesterol. In atherosclerosis, 7-ketocholesterol and 7*β*-hydroxycholesterol are the most abundant oxysterols observed in macrophage-derived lipid-rich foam cells following 27-hydroxycholesterol. 7-Ketocholesterol has been attributed to the inhibition of cholesterol export from cells, induction of apoptosis, altered endothelial permeability, inhibition of nitric oxide release, and disruption of Ca^2+^ fluxes [4]. In the present study, plasma 7-ketocholesterol concentration was the only oxysterol that remained unchanged in the obese mice. Furthermore, 7-ketocholesterol remained unchanged in the liver and adipose tissue. Lyons et al. [42] demonstrated that 7-ketocholesterol is rapidly metabolized by the liver following its administration in rats by its reduction to 7*β*-hydroxycholesterol by 11*β*-hydroxysteroid dehydrogenase type 1 (11*β*-HSD1) [43]. 7-Ketocholesterol is also a substrate for CYP27A1, which is expressed in the liver and several extrahepatic tissues and macrophages [44]. We observed large increases in plasma 7*β*-hydroxycholesterol (2.4-fold) and 27-hydroxycholesterol (3.9-fold) and it is possible that 7-ketocholesterol was rapidly cleared by one or both metabolic pathways.

In contrast to 7-ketocholesterol, 7*α*-hydroxycholesterol and 7*β*-hydroxycholesterol were significantly elevated in the plasma; however, none of the 7-oxycholesterol oxysterol levels were altered in the liver or adipose tissue. 7*α*-Hydroxycholesterol is the primary intermediate for the bile acid synthesis pathway in the liver. Gnerre et al. [31] showed that, with increased hepatic cholesterol, CYP7A1 mRNA increased via LXR*α* activation. In contrast, LXR does not activate CYP7A1 in humans but may be regulated by farnesoid-X-receptor signaling [45]. Interestingly, overexpression of hepatic CYP7A1 in mice has been shown to protect mice from high-fat diet-induced obesity, insulin resistance, and hepatic steatosis and maintain cholesterol, bile acid, and triglyceride homeostasis [46]; however, metabolic syndrome induced by a high-fat sucrose diet in rats reduced CYP7A1 mRNA expression and protein mass [47]. In the present study, despite elevated hepatic cholesterol observed in the obese mice, CYP7A1 mRNA was unchanged, which may in part explain the unchanged hepatic levels of 7*α*-hydroxycholesterol in these obese mice. It is plausible that the obesity and subsequent NAFLD in our mice may have inhibited the upregulation of LXR and CYP7A1 activities.

Compared to the other oxysterols measured in this study, less is known about the function and metabolism of epoxycholesterol oxysterols. In patients with hypercholesterolemia, increased serum 5,6*α*-epoxycholesterol concentrations were observed with a significant correlation between the degree of atherosclerosis [48]. Similar to 7*α*-hydroxycholesterol, 5,6*α*-epoxycholesterol and 5,6*β*-epoxycholesterol are produced by the peroxidation and autooxidation of cholesterol [49]. In addition, 5,6*α*-epoxycholesterol has been shown in vitro to be an antagonist of LXR [50]. LXR*α* and LXR*β* are oxysterol dependent transcription factors that induce the expression of genes involved with cholesterol efflux and transport and decrease the expression of inflammatory mediators such as nuclear factor-*κ*B [51, 52]. Therefore, 5,6*α*-epoxycholesterol may serve as an important mediator for the development of atherosclerosis and related inflammatory diseases. Circulating 5,6*α*-epoxycholesterol and 5,6*β*-epoxycholesterol concentrations were 1.6- and 2.5-fold higher, respectively, in the obese mice. In addition, 5,6*β*-epoxycholesterol and 5,6*α*-epoxycholesterol levels in adipose tissue were 64.6% and 137.9% greater, respectively, although not significant due in part to the large variability observed in the obese mice. Interestingly, the hepatic epoxycholesterol oxysterols, as well as the 7-oxygenated oxysterols, remained unchanged in the obese mice. This is likely due to the observed increase in hepatic SULT2B1b mRNA expression in the obese mice. The SULT2B1b isoenzyme is a cytosolic sulfotransferase, which sulfonates steroids and sterols to prevent their cytotoxic effects. The oxysterol substrates for SULT2B1b include 5,6*α*-epoxycholesterol, 5,6*β*-epoxycholesterol, 7-ketocholesterol, 7*α*-hydroxycholesterol, 7*β*-hydroxycholesterol, 27-hydroxycholesterol, and 7*α*-hydroperoxide derivatives [53, 54]. Therefore, it is plausible that SULT2B1b mRNA expression was upregulated in mice on HFC diet in order to prevent accumulation of possible cytotoxic oxysterols in the liver.

## 5. Conclusions

In summary, we have demonstrated that obesity induced by a HFC diet resulted in markedly increased concentrations of both enzymatically and nonenzymatically derived oxysterols in the circulation as well as hepatic and adipose tissues. The increased circulating oxysterols were associated with increased cholesterol, triglyceride, and phospholipid in all lipoprotein fractions and the presence of the LDL/HDL_1_ phenotype typically observed in obese mice. Despite the increase in hepatic and adipose tissue oxysterols, we did not observe a change in the expression of hepatic CYP7A1, 3A11, or 27A1 mRNA and CYP27A1 mRNA in adipose tissue. We did detect the presence of SULT2B1b mRNA in the adipose tissue and an increase in hepatic SULT2B1b mRNA expression in the obese mice, possibly as a compensatory mechanism to prevent the accumulation of these cytotoxic oxysterols in these tissues. To our knowledge, this study is first to characterize the metabolism of oxysterols in diet-induced obesity. Future studies using this diet-induced obesity mouse model are needed to elucidate the role of increased oxidative stress on the modulation of oxysterol metabolism.

## Figures and Tables

**Table 1:** Post-diet lipids in liver tissue and adipose tissue.

Variables	Liver tissue		Adipose tissue	
	ND (*n* = 7)	HFC (*n* = 9)	ND (*n* = 8)	HFC (*n* = 10)
Total cholesterol (mg/mg protein)	0.10 ± 0.01	0.33 ± 0.06^‡^	4.04 ± 2.23	8.17 ± 3.07^†^
Triglyceride (mg/mg protein)	0.37 ± 0.05	1.49 ± 0.12^‡^	32.81 ± 16.10	59.96 ± 22.72*

*Note*. Data are presented as mean ± SD. *P < 0.05, ^†^ *P* < 0.01, and ^‡^ *P* < 0.001 significantly different than control group.

**Table 2:** The effects of the high fat/high cholesterol diet on fasting lipoprotein lipid composition.

Variables	VLDL		LDL		LDL/HDL_1_		HDL	
	ND	HFC	ND	HFC	ND	HFC	ND	HFC
Cholesterol (mg/dL)	0.79 ± 0.41	6.36 ± 1.84^†^	1.24 ± 0.14	4.29 ± 2.32*	1.39 ± 0.56	9.42 ± 7.26	17.56 ± 5.05	38.04 ± 11.60*
Triglyceride (mg/dL)	0.89 ± 0.84	1.66 ± 0.95	0.24 ± 0.14	2.52 ± 1.71*	0.31 ± 0.21	4.43 ± 3.15*	8.70 ± 2.11	18.41 ± 5.82*
Phospholipid (mg/dL)	1.19 ± 0.84	1.58 ± 0.95	0.42 ± 0.14	1.93 ± 0.92*	0.39 ± 0.31	3.90 ± 2.78*	19.78 ± 7.04	39.06 ± 14.53*
Protein (mg/dL)	5.10 ± 0.86	2.62 ± 0.70^†^	2.30 ± 1.16	3.52 ± 1.27	1.91 ± 0.92	5.93 ± 2.62*	46.43 ± 10.76	43.73 ± 9.24
Cholesterol (µg/µg protein)	0.19 ± 0.06	2.41 ± 0.30^‡^	0.98 ± 1.14	1.15 ± 0.23	1.12 ± 1.07	1.42 ± 0.48*	0.38 ± 0.04	0.86 ± 0.12^‡^
Triglyceride (µg/µg protein)	0.23 ± 0.17	0.60 ± 0.32	0.16 ± 0.16	0.64 ± 0.30*	0.32 ± 0.45	0.67 ± 0.24	0.19 ± 0.01	0.41 ± 0.09^†^
Phospholipid (µg/µg protein)	0.29 ± 0.10	0.59 ± 0.15^†^	0.24 ± 0.16	0.54 ± 0.23*	0.25 ± 0.19	0.61 ± 0.15*	0.41 ± 0.08	0.87 ± 0.24*

*Note*. Data are presented as mean ± SD. *P < 0.05, ^†^ *P* < 0.01, and ^‡^ *P* < 0.001 significantly different than control group. *n* = 6 per group. HFC: high fat/high cholesterol diet group; VLDL: very-low density lipoprotein; LDL: low-density lipoprotein; sdLDL/HDL_1_: small, dense low-density lipoprotein/high-density lipoprotein-1; HDL: high-density lipoprotein.

**Table 3:** Oxysterol levels in control and high fat-cholesterol diets.

Variables	ND	HFC
4*β*-OH	0.10 ± 0.01	0.17 ± 0.06
5,6*β*-Epoxy	0.36 ± 0.05	0.60 ± 0.35
5,6*α*-Epoxy	0.34 ± 0.05	0.82 ± 0.51
7-keto	0.84 ± 0.04	0.72 ± 0.08*
7*α*-OH	0.64 ± 0.47	0.94 ± 0.12
7*β*-OH	1.09 ± 0.06	0.95 ± 0.50
25-OH	0.21 ± 0.18	0.37 ± 0.05
27-OH	0.02 ± 0.01	1.95 ± 0.20^†^

*Note*. Data are present as mean ± SD, *n* = 5 per group. *P < 0.05, ^†^ *P* < 0.001. Oxysterols are presented as ng/mg of chow. 7*α*-OH: 7*α*-hydroxycholesterol; 7*β*-OH: 7*β*-hydroxycholesterol; 7-keto: 7-ketocholesterol; 4*β*-OH: 4*β*-hydroxycholesterol; 5,6*α*-epoxy: 5,6*α*-epoxycholesterol; 5,6*β*-epoxy: 5,6*β*-epoxycholesterol; 25-OH: 25-hydroxycholesterol; 27-OH: 27-hydroxycholesterol.

**Table 4:** Post-diet oxysterols in plasma, liver tissue, and adipose tissue.

Variables	Plasma (ng/mL)		Liver tissue (ng/mg protein)		Adipose tissue (ng/mg protein)	
	ND	HFC	ND	HFC	ND	HFC
4*β*-OH	6.8 ± 0.7	67.0 ± 9.6^‡^	7.2 ± 0.9	18.3 ± 7.7*	17.4 ± 8.2	130.9 ± 62.2^†^
5,6*β*-epoxy	3.8 ± 0.4	10.0 ± 3.0*	12.3 ± 5.3	11.1 ± 3.2	49.7 ± 36.4	81.8 ± 50.2
5,6*α*-epoxy	2.8 ± 0.6	10.0 ± 4.1*	7.8 ± 2.7	9.2 ± 2.4	33.7 ± 21.1	80.2 ± 67.5
7-keto	1.2 ± 0.3	1.9 ± 0.3	7.2 ± 3.5	7.2 ± 3.0	62.7 ± 41.5	74.6 ± 46.5
7*α*-OH	1.2 ± 0.1	3.2 ± 1.2*	5.4 ± 2.0	4.4 ± 1.5	36.9 ± 21.7	34.8 ± 11.6
7*β*-OH	1.0 ± 0.1	2.4 ± 0.5*	3.2 ± 1.1	3.8 ± 1.3	35.1 ± 25.6	28.3 ± 9.2
25-OH	0.6 ± 0.4	0.7 ± 0.1	5.8 ± 7.4	18.5 ± 30.6	146.5 ± 107.7	52.1 ± 57.1
27-OH	0.7 ± 0.1	2.6 ± 0.3^‡^	1.3 ± 0.4	1.9 ± 0.6	18.3 ± 7.5	45.8 ± 16.1^†^

*Note*. Data are presented as mean ± SD. *P < 0.05, ^†^ *P* < 0.01, ^‡^ *P* < 0.001 significantly different than control group. *n* = 6 per group. 5,6*α*-epoxy: 5*α*,6*α*-epoxycholesterol; 5,6*β*-epoxy: 5*β*,6*β*-epoxycholesterol; 4*β*-OH: 4*β*-hydroxycholesterol; 25-OH: 25-hydroxycholesterol; 27-OH: 27-hydroxycholesterol; 7*α*-OH: 7*α*-hydroxycholesterol; 7*β*-OH: 7*β*-hydroxycholesterol; 7-keto: 7-ketocholesterol.
